# A Dual-Channel Supply Chain Coordination under Carbon Cap-and-Trade Regulation

**DOI:** 10.3390/ijerph15071316

**Published:** 2018-06-23

**Authors:** Qi Qi, Jing Wang, Jianteng Xu

**Affiliations:** 1 School of Economics and Management, Beihang University, Beijing 100191, China; jim08@buaa.edu.cn; 2 School of Management, Qufu Normal University, Rizhao 276826, China; jiantengxu@163.com

**Keywords:** dual-channel, cap-and-trade, price discount, Stackelberg game

## Abstract

We consider a dual-channel supply chain coordination under a carbon cap-and-trade regulation. The dual-channel refers to the traditional retail channel and the network direct channel, and both two channels’ selling prices can affect the market demand. We formulate the problem as a supplier-Stackelberg game model and obtain the optimal pricing decisions and corresponding profits in centralized and decentralized systems. We explore the effects of cap-and-trade regulation on optimal decisions and profits. To improve the performance of the decentralized system, we propose online channel price discount and offline channel price discount contracts to coordinate dual-channel supply chain and provide a transfer payment mechanism to make win-win of both sides. Moreover, we investigate how carbon regulation affects the coordination performance. Numerical examples illustrate the process to find the appropriate price discount coefficient and show the coordination effects of two contracts.

## 1. Introduction

There are some new words in today’s world of e-business, including direct which refers to customers buying products through the network and then products are sent out from supplier or manufacturer. In contrast to direct, we have a better understanding of retail which refers to customers being able to purchase and return product in retail shops. Now, many firms are selling their products through both traditional retail (offline) and internet (online) channels, such as Panasonic, Lenovo and Estee Lauder [1,2], which is called dual-channel distributing system. The emergence of the dual-channel brings new opportunities and challenges to enterprises. On the one hand, direct channel extends the market to the world and attract more new consumers; on the other hand, adding new channels could affect current price and retail channel demand and has a negative effect on the retail channel. Hence it is very important and significant to research the pricing strategies and coordination mechanism of a dual-channel supply chain system.

In addition, modern society has been giving an increasing attention to environmental protection, and it is becoming more and more important to decrease emissions of greenhouse gasses, especially the emission of carbon dioxide. The Intergovernmental Panel on Climate Change (IPCC) reported that human interference in climate is taking place as the rapid development of economic [3]. As more and more greenhouse gases produced by human activities are released into the atmosphere, the greenhouse effect is increasingly severe. Some counties and organizations have developed regulations to control carbon emission, including cap-and-trade, carbon tax regulations. Cap-and-trade regulation is market-based and regard as a efficient mechanism to control carbon emissions. In 2008, the first global trading platform for carbon emission began to run. At present, there are more than 20 carbon trading platforms in the world, including European Union Greenhouse Gas Emission Trading Scheme, UK Emissions Trading Group, Chicago Climate Exchange and National Trust of Australia. Chinese government implemented cap-and-trade regulation in seven plot regions in 2013 and has launched a national carbon emissions trading system in 2017. In this context, how do dual-channel agents adjust operational decisions in order to control carbon emission? How does a cap-and-trade regulation effect operational decisions of dual-channel supply chain? Is there a contract to coordinate the dual-channel supply chain under cap-and-trade regulation? These questions are all our focus.

In this paper, we investigate a dual-channel supply chain under carbon cap-and-trade regulation. We first analyze optimal wholesale price and retail prices of two channels in centralized and decentralized systems and compare profits of two systems. Then we propose two price discount contracts, online channel price discount contract and offline channel price discount, to coordinate the dual-channel supply chain and compare the coordination effects of two contracts. This paper contributes to the literature on dual-channel supply chain in several aspects. First, it explores the affects of cap-and-trade regulation on price decisions in a dual-channel supply chain. Second, it illustrates the coordination effect of two price discount contracts and describes a transfer payment mechanism to achieve win-win. These results could guide enterprises in making operational decisions.

The reminder of this paper is organized as follows. Section 2 summarizes the related literature. Section 3 describes the problem and presents further assumptions and notations. Section 4 solves the optimal pricing with the supplier Stackelberg game and obtains corresponding optimal profits in centralized and decentralized systems respectively. Section 5 presents two kinds of price discount contracts to coordinate the supply chain. Conclusion and future research are showed in Section 6.

## 2. Literature Review

Our paper is related to two streams of research, dual-channel supply chain management and supply chain management under carbon emission regulations.

### 2.1. Dual-Channel Supply Chain Management

With the development of e-commerce, more and more literature have focused on the operational decisions of dual-channel supply chain. Rhee and Park divided consumers into two groups: price sensitive and service sensitive. They found that the dual-channel is better when their valuations of retail services across channels are similar. Some scholars have also researched dual-channel supply chain, these literature could roughly divided two types. One is about pricing and other operational decisions in dual-channel supply chain [4,5,6]; the other is about pricing and coordination of dual-channel supply chain which will be detailed reviewed since it is most related to this paper.

There is a range of research on coordination of dual-channel supply chain. Cai [7] studied four different channel structures of two-echelon supply chain, including two single-channel supply chain and two dual-channel supply chain. They compared their performances with and without coordination, and found that the preference of supplier and retailer varies with parameters such as demand, cost and substitutability. Chen et al. [8] presented the conditions under which both supplier and retailer prefer a dual-channel supply chain when the supplier is a leader of Stackelberg game, and illustrated how a complementary agreement contract brought a win-win result. David and Adida [9] researched a supply chain in which a single supplier sells products through both a direct channel and symmetric retailers. They proved that a linear quantity discount contract can coordinate a dual-channel supply chain. However, there is a little literature considering the pricing and coordination. Dumrongsiri et al. [10] consumed that consumers choose buying channel through price and service, and studied the optimal pricing of direct channel and the optimal price and ordering quantity of retailer. They provided the equilibrium conditions under which the supplier and retailer could share the market. Cai et al. [11] considered the pricing strategies and effects of price discount contract of dual-channel supply chain. He analyzed the Stackelberg game with both supplier dominated and retailer dominated, and Nash game, respectively. He found that price discount contract outperform the non-contract. Liu et al. [12] considered the dual-channel supply chain with price-dependent stochastic demand and asymmetric information. They studied the optimal production and pricing strategies of dual-channel supply chain, and designed two kinds of contracts to coordinate the decentralized system. Xu et al. [13] researched the coordination of a dual-channel supply chain with the risk-averse members based on mean-variance model. They proved that the two-way revenue sharing contract can bring a win-win results. Above literature studied the dual-channel supply chain in a general circumstance, that is, there are no external constraints of environment protection regulations on the supply and decision makers.

### 2.2. Supply Chain Management under Carbon Emission Regulations

In recent decades, however, as the awareness of environmental protection increasing, some scholars have begun to focus on decision making of firms in the context of environmental protection [14,15,16,17,18]. With the increasing attention to carbon emission reduction, some scholars have studied the impact of carbon regulations on business decision. Hua et al. [19] and Chen et al. [20] introduced carbon regulations, such as carbon cap, carbon tax and carbon cap-and-trade, into the classical Economic Order Quantity (EOQ), and compared the optimal order quantity and lowest total cost before and after introducing carbon regulations. Toptal et al. [21] further studied the joint production and investment strategy of carbon emission reduction under carbon cap, carbon tax and carbon cap-and-trade regulations. Zhang et al. [22], based on the newsvendor model, researched how to balance to achieve maximizing expected profit when firms obtain emission permits through three ways, emission quota, cap-and-trade and cleaning treatment. Song et al. [23] studied the classical newsvendor model under carbon cap, carbon tax and cap-and-trade regulations. They assumed the selling price is exogenous variable, and compared the impact of three carbon regulations on carbon emission and total profit when the demand obeys the normal distribution. Dong et al. [24] studied the retailer’s order quantity and manufacturer’s sustainable investment of centralized and decentralized systems in a two-echelon supply chain under cap-and-trade regulation. They assumed the demand is affected by sustainable investment and the price is exogenous variable. Bai et al. [25] considered both emission reduction investment and promotional effort under cap-and-trade regulation, and they proposed two contracts to coordinate the supply chain. They found that coordination could result in fewer carbon emissions and more profit. Song et al. [26] explored a capacity expansion problem under carbon regulations, and they found that high tax rate and low tax rate had different effects on optimal decisions. Xu et al. [27] considered a newsvendor model which has partial demand information and investigated two distributionally robust models under two carbon regulations. They found that the demand information parameters have more effect on optimal worst-case expected profit.

The above literature researched decision-making and coordination of low-carbon supply chain; however, they did not consider the operation management take into account environment based on dual-channel supply chain. As far as we know, some literature focussed on the impact of environmental protection on the dual-channel supply chain. Modak et al. [28] considered the consumer surplus as a form of corporate social responsibility(CSR) effect, and incorporate it into the dual-channel supply chain. They found that when supplier and retailer concentrate more on CSR, their profits are higher than their individual profits. Li et al. [29] incorporate the greening strategies into the dual-channel supply chain, and studied the pricing policies in centralized and decentralized systems. They provided the conditions in which the manufacturer would prefer to open a direct channel, and found that the retail price in centralized system is higher than that in decentralized system. However, they did not analyze the effects of carbon regulations on operation decisions of dual-channel supply chain. Different from this literature, this paper considers the pricing and coordination of the dual-channel supply chain under cap-and-trade regulation which is imposed by government.

## 3. Description and Notations

**Figure 1:**
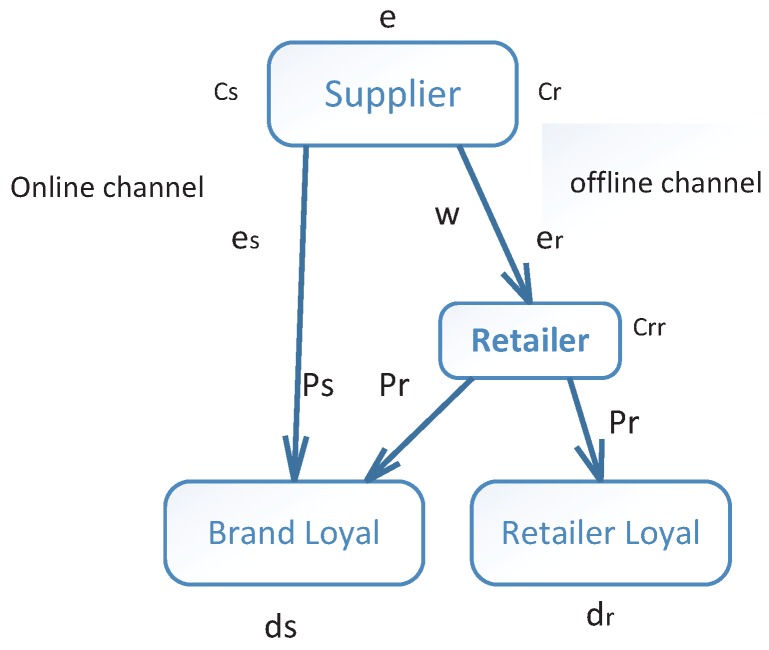
The two-echelon supply chain with online and offline selling channels.

This study considers a two-echelon dual-channel supply chain coordination under cap-and-trade regulation. The supplier sells a certain product through online and offline channels. In the offline channel, that is a traditional retail channel, the supplier sells product at price $p_{r}$, and in the online channel, the supplier sells product directly to consumers through Internet at price $p_{s}$. Consumers are free to choose purchase channels. Some consumers prefer to purchase products only from the retailer, and the other purchase products from both the supplier and retailer. So they can be divided into two types, retailer loyal and brand loyal [30]. When the selling prices of online and offline are equal, we assume θ of brand loyal consumers prefer to purchase directly from the supplier, that is the initial proportion of brand loyal consumers preferring the online channel is θ, and the initial proportion of brand loyal consumers preferring the offline channel is 1−θ. While some consumers will change their preference of purchase channel with price-sensitive parameter η because of the gap of two channel selling prices [31]. We also assume that the supplier follows the “make-to-order” policy and the retailer follows the “lot-for-lot” policy, which are common assumptions in the literature on supply chain management. The main process of carbon emissions contains two parts, the production of supplier and procurement of two channels. The supplier is responsible for the emissions related to production and procurement in online channel, and the retailer is responsible for the emissions related to procurement in offline channel [32]. The dual-channel supply chain is illustrated in Figure 1. The major notations used in this paper are listed in Table 1.

Similar to some existing literature [30,31], we assume that the demand of unit market size is a function of the channel selling price with an elasticity parameter β. Then the demand functions of online and offline channels can be described as follows:

$$\begin{array}{ccc} D_{s}\left(p_{s},p_{r}\right) & = & d_{s}\left[\theta \left(1-\beta p_{s}\right)-\eta \left(p_{s}-p_{r}\right)\right]\\ & = & d_{s}\eta p_{r}-d_{s}\left(\theta \beta +\eta \right)p_{s}+d_{s}\theta \\ D_{r}\left(p_{s},p_{r}\right) & = & d_{r}\left(1-\beta p_{r}\right)+d_{s}\left[\left(1-\theta \right)\left(1-\beta p_{r}\right)+\eta \left(p_{s}-p_{r}\right)\right]\\ & = & d_{s}\eta p_{s}-Ap_{r}+d_{r}+d_{s}\left(1-\theta \right) \end{array}$$

In the rest of this paper, the superscript “*” is added to the relative variables to represent their corresponding optimal values. The subscript “c” is added to the relative variables to represent the values in the centralized system, subscript “*r*” and “*s*” are added to the relative variables to represent the values with offline channel and online channel price discount contracts, respectively. For convenience of calculation, let $D_{s}$ and $D_{r}$ denote $D_{s}\left(p_{s},p_{r}\right)$ and $D_{r}\left(p_{s},p_{r}\right)$, respectively. Set $A=d_{r}\beta +d_{s}\left(1-\theta \right)\beta +d_{s}\eta$ and $Z=\frac{d_{s}^{2}\eta^{2}}{A}-d_{s}\left(\theta \beta +\eta \right)$, we have A>0,Z<0.

## 4. Model Analysis

To reveal how cap-and-trade regulation affects the selling price of online and offline channel under different decision systems, in this section, we will model and compare the decentralized and centralized systems.

### 4.1. Model Analysis for the Centralized System

Total carbon emissions $E_{c}\left(p_{s},p_{r}\right)$ in the centralized system is

$$\begin{array}{ccc} E_{c}\left(p_{s},p_{r}\right) & = & \left(D_{r}+D_{s}\right)e+D_{s}e_{s}+D_{r}e_{r}\\ & = & D_{r}e_{2}+D_{s}e_{1},\;where\;e_{1}=e+e_{s},e_{2}=e+e_{r} \end{array}$$

In centralized decision system, the supplier and retailer determine the selling price of two channels together. The total profit of centralized system is given by

$$\begin{array}{ccc} \Pi_{c}\left(p_{s},p_{r}\right) & = & D_{s}\left(p_{s}-c_{s}\right)+D_{r}\left(p_{r}-c_{r}-c_{rr}\right)-c_{e}\left(D_{r}e_{2}+D_{s}e_{1}-K_{c}\right)\\ & = & d_{s}\theta \left(1-\beta p_{s}\right)\left(p_{s}-c_{s}-c_{e}e_{1}\right)+\left[d_{r}+d_{s}\left(1-\theta \right)\right]\left(1-\beta p_{r}\right)\left(p_{r}-c_{r}-c_{rr}-c_{e}e_{2}\right)\\ & & -d_{s}\eta \left(p_{s}-p_{r}\right)\left(p_{s}-p_{r}-c_{s}+c_{rr}+c_{r}-c_{e}e_{1}+c_{e}e_{2}\right)+c_{e}K_{c} \end{array}$$

On the right-hand side of formula above, the first item is the sales revenue by online channel, the second is the sales revenue by offline channel, and the last is the cost (revenue) caused by cap-and-trade regulation. When the emissions of system is higher (lower) than the capacity assigned by government, a cap-and-trade regulation means a cost (revenue) for system.

**Corollary** **1.** *There exists uniquely optimal solutions in the centralized decision system.*

**Proof.**  Please see Appendix A. ☐

From Corollary 1 we can obtain the following conclusion.

**Theorem** **1.** *In centralized system, two optimal selling prices of two channels as follows*

$$\begin{array}{ccc} p_{s,c}^{*} & = & \frac{1+\beta c_{s}}{2\beta }+\frac{c_{e}e_{1}}{2}\\ p_{r,c}^{*} & = & \frac{1+\beta (c_{r}+c_{rr})}{2\beta }+\frac{c_{e}e_{2}}{2} \end{array}$$

**Proof.**  Please see Appendix B. ☐

It is worth noting that Theorem 1 is conditional on $1-\beta (c_{s}+c_{e}e_{1})\geq 0$ and $\theta \left(1-\beta \left(c_{s}+c_{e}e_{1}\right)\right)>\eta \left(c_{s}-c_{r}-c_{rr}+c_{e}\left(e_{s}-e_{r}\right)\right)$, otherwise the market demand of online channel is negative. Moreover, we find that the optimal retail prices are depend on carbon emissions and the unit price of carbon trade. Specifically,

(i) the higher carbon emissions is, the higher two retail prices are. That is high carbon emissions could lead to high retail prices which is disadvantageous for the market demand.
(ii) the unit price of carbon trade is positively correlated with retail prices. That is the retail prices would also be high due to the unit price of carbon trade.

Therefore, it is necessary to reduce carbon emissions for firm in order to ensure the market demand, and it also shows that cap-and-trade regulation is effective in controlling carbon emissions. In addition, we find that the optimal retail prices is not depend on the carbon capacity. Next, we explore the corresponding optimal profit under cap-and-trade regulation.

From Theorem 1 we can have the following result.

**Corollary** **2.** *In centralized system, the optimal total profit of whole supply chain as follows*

$$\begin{array}{ccc} \Pi_{c}^{*} & = & d_{s}\theta \beta {\left(\frac{1-\beta c_{s}}{2}-\frac{c_{e}e_{1}}{2}\right)}^{2}+\left[d_{r}+d_{s}\left(1-\theta \right)\right]\beta {\left(\frac{1-\beta c_{r}-\beta c_{rr}}{2\beta }-\frac{c_{e}e_{2}}{2}\right)}^{2}\\ & & +d_{s}\eta {\left(\frac{c_{s}-c_{r}-c_{rr}}{2}+\frac{c_{e}\left(e_{s}-e_{r}\right)}{2}\right)}^{2}+c_{e}K_{c} \end{array}$$

We can find that the optimal profit is dependent on the unit price of carbon trade and the emission capacity. If total carbon emissions lower the capacity assigned to dual-channel supply chain, the system can sell the remaining carbon capacity through carbon market and will obtain corresponding profit, while if total carbon emissions higher the capacity assigned to dual-channel supply chain, the system have to buy the remaining carbon capacity through carbon market and will pay out corresponding cost.

### 4.2. Model Analysis for the Decentralized System

In reality, agents make their own decisions separately in most supply chains. Hence we model the decentralized system as a supplier-lead Stackelberg game, in which the decision process is as follows. The supplier first determines the wholesale price and the selling price in online channel, then the retailer determines the selling price of offline channel with the wholesale price declared by the supplier, a setting used in [11]. In decentralized system, carbon emissions of supplier and retailer are $\left(D_{r}+D_{s}\right)e+D_{s}e_{s}=D_{s}e_{1}+D_{r}e$ and $D_{r}e_{r}$, respectively, where $e_{1}=e+e_{s}$.

The profit functions of supplier and retailer as follows

$$\begin{array}{ccc} \Pi_{s}\left(p_{s},w\right) & = & D_{s}\left(p_{s}-c_{s}\right)+D_{r}\left(w-c_{r}\right)-c_{e}\left(D_{s}e_{1}+D_{r}e-K_{s}\right)\\ & = & \left(d_{s}\theta -d_{s}\left(\theta \beta +\eta \right)p_{s}+d_{s}\eta p_{r}\right)\left(p_{s}-c_{s}-c_{e}e_{1}\right)+(d_{r}+d_{s}\left(1-\theta \right)\\ & & +d_{s}\eta p_{s}-Ap_{r})\left(w-c_{r}-c_{e}e\right)+c_{e}K_{s}\\ \Pi_{r}\left(p_{r}\right) & = & D_{r}\left(p_{r}-w-c_{rr}\right)-c_{e}\left(D_{r}e_{r}-K_{r}\right)\\ & = & \left(d_{r}+d_{s}\left(1-\theta \right)+d_{s}\eta p_{s}-Ap_{r}\right)\left(p_{r}-w-c_{rr}-c_{e}e_{r}\right)+c_{e}K_{r} \end{array}$$

It is easy to verify that $\pi_{r}\left(p_{r}\right)$ is a convex programming about $p_{r}$, which implies that optimal solution of $\pi_{r}\left(p_{r}\right)$ exists uniquely. Then by solving $\frac{\partial \pi_{r}\left(p_{r}\right)}{\partial p_{r}}=0$, we can obtain the optimal of $\pi_{r}\left(p_{r}\right)$

(1) $$\begin{array}{c} p_{r}\left(p_{s},w\right)=\frac{d_{s}\eta }{2A}p_{s}+\frac{w+c_{rr}+c_{e}e_{r}}{2}+\frac{d_{r}+d_{s}\left(1-\theta \right)}{2A} \end{array}$$

Substituting Equation (1) to $\Pi_{s}\left(p_{s},w\right)$, we can obtain following result.

**Corollary** **3.** *There exists uniquely optimal solutions of $\Pi_{s}\left(p_{s},w\right)$.*

**Proof.**  Please see Appendix C. ☐

Then we can obtain the following results.

**Theorem** **2.** *The optimal solutions and corresponding profit in decentralized system as follows*

$$\begin{array}{ccc} p_{s}^{*} & = & \frac{1+\beta c_{s}}{2\beta }+\frac{c_{e}e_{1}}{2}\\ w^{*} & = & \frac{1+\beta (c_{r}-c_{rr})}{2\beta }+\frac{c_{e}\left(e-e_{r}\right)}{2}\\ p_{r}^{*} & = & \frac{3+\beta (c_{r}+c_{rr})}{4\beta }-\frac{d_{s}\eta \left(1-\beta c_{s}\right)}{4A\beta }+d_{s}\eta \frac{c_{e}e_{1}}{4A}+\frac{c_{e}e_{2}}{4} \end{array}$$

**Proof.**  Please see Appendix D. ☐

It is worth noting that Theorem 1 is conditional on $1-\beta (c_{s}+c_{e}e_{1})\geq 0$ and $2A\theta \left(1-\beta \left(c_{s}+c_{e}e_{1}\right)\right)>\eta \left(A\left(2c_{s}-c_{r}-c_{rr}\right)+\left(2A-d_{s}\eta \right)c_{e}e_{1}-c_{e}e_{2}-d_{r}-d_{s}\left(1-\theta \right)-d_{s}\eta c_{s}\right)$, otherwise the market demand of online channel is negative.

Moreover, we can find that the optimal wholesale price and retail prices are depend on carbon emissions and the unit price of carbon trade. In particularly,

(i) the higher carbon emissions during production and transportation are, the higher two optimal retail prices are. That is high carbon emissions could lead to high retail prices which is disadvantageous for the market demand.
(ii) For optimal wholesale price, the higher emissions during production is, the higher the optimal wholesale price is, while the higher the emissions during transportation for offline channel is, the lower the optimal wholesale price is.
(iii) the unit price of carbon trade is positively correlated with both optimal wholesale price and retail prices. That is the unit price of carbon trade could increase the firm’s cost and so improve the wholesale price and retail prices.

Therefore, it also shows that cap-and-trade regulation is effective in controlling carbon emissions in decentralized system. In addition, we find that both the optimal wholesale price and retail prices are not depend on the carbon capacity. Next we explore the corresponding optimal profit of retailer and supplier under cap-and-trade regulation.

**Corollary** **4.** *In decentralized system, the optimal profits of retailer and supplier as follows*

$$\begin{array}{ccc} \Pi_{r}^{*} & = & \left[\frac{A\left(1-\beta c_{r}-\beta c_{rr}\right)-d_{s}\eta \left(1-\beta c_{s}\right)}{4\beta }+d_{s}\eta \frac{c_{e}e_{1}}{4}-A\frac{c_{e}e_{2}}{4}\right]\\ & & \cdot \left[\frac{A\left(1-\beta c_{r}-\beta c_{rr}\right)-d_{s}\eta \left(1-\beta c_{s}\right)}{4A\beta }+d_{s}\eta \frac{c_{e}e_{1}}{4A}-\frac{c_{e}e_{2}}{4}-\frac{c_{e}\left(e-e_{r}\right)}{2}\right]+c_{e}K_{r}\\ \Pi_{s}^{*} & = & [d_{s}\theta \frac{1-\beta c_{s}}{2}-d_{s}\eta \frac{2\beta c_{s}-1-\beta c_{r}-\beta c_{rr}}{4\beta }-d_{s}^{2}\eta^{2}\frac{1-\beta c_{s}}{4A\beta }-\left(d_{s}\theta \beta +d_{s}\eta \right)\frac{c_{e}e_{1}}{2}\\ & & +d_{s}^{2}\eta^{2}\frac{c_{e}e_{1}}{4A}+d_{s}\eta \frac{c_{e}e_{2}}{4}]\cdot \left(\frac{1-\beta c_{s}}{2\beta }-\frac{c_{e}e_{1}}{2}\right)+[\frac{A\left(1-\beta c_{r}-\beta c_{rr}\right)-d_{s}\eta \left(1-\beta c_{s}\right)}{4\beta }\\ & & +d_{s}\eta \frac{c_{e}e_{1}-Ac_{e}e_{2}}{4}]\cdot \left(\frac{1-\beta c_{r}-\beta c_{rr}}{2\beta }-\frac{c_{e}e_{2}}{2}\right)+c_{e}K_{s} \end{array}$$

We can find that the optimal profit is dependent on the unit price of carbon trade and the emission capacity. If total carbon emissions lower the capacity assigned to supplier (or retailer), the supplier (or retailer) can sell the remaining carbon capacity through carbon market and will obtain corresponding profit, while if total carbon emissions higher the capacity, the supplier (or retailer) have to buy the remaining carbon capacity through carbon market and will pay out corresponding cost.

Compare with the situation that there are no regulations, we can obtain the following results.

**Corollary** **5.** *The optimal sell prices under cap-and-trade regulation are higher than those without regulations, while the wholesale price and the corresponding system profit depends on some parameters.*

We can find that cap-and-trade regulation would improve the sell prices of dual-channel supply chain, while the change of optimal wholesale price is related to the carbon emissions during production of supplier and transportation of offline channel. Moreover, the profits of supplier and retailer also depend on the parameters of carbon regulation, such as carbon capacity assigned by government and unit price of carbon trade. When the government gives a high carbon capacity, the supplier or retailer can obtain extra profit by selling remaining carbon credits and the profit may be increase.

Due to the complexity of these formulas, we use a numerical example to show the effect of carbon regulation on optimal decisions and profits under centralized and decentralized systems. Let $d_{r}=d_{s}=200,\beta =0.01,\theta =0.5,\eta =0.005,c_{r}=10,c_{rr}=3,c_{s}=12,K_{r}=5000,K_{s}=8960$, $e_{r}=e_{s}=5,e=3,c_{e}=0-11$.

**Figure 2:**
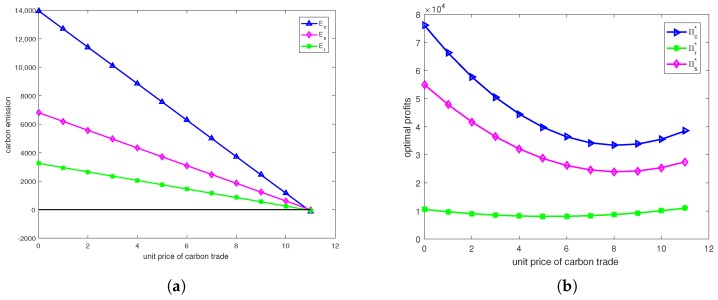
(**a**) The effects of unit price of carbon trade on emission. (**b**) The effects of unit price of carbon trade on profit.

From Figure 2 we can find that cap-and-trade regulation could effectively reduce carbon emissions, and the higher the unit price of carbon trade, the more obvious the reduction is, but we should note that the unit price of carbon trade should not be too high. Moreover, we find that the optimal profit would decrease first and then increase. That is because the higher unit price of carbon trade could lead to fewer carbon emissions as shown in Figure 2a, and the firm would sell extra carbon credit to obtain profit.

### 4.3. Comparison between Centralized and Decentralized Systems

**Figure 3:**
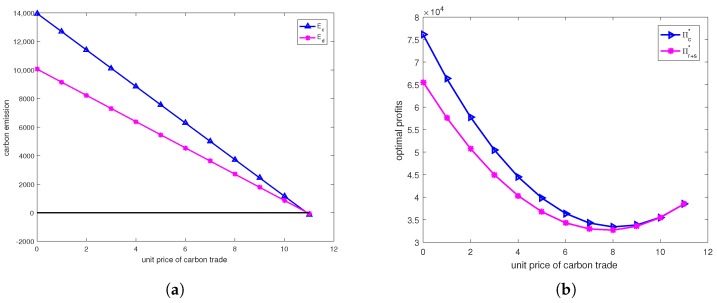
(**a**) The effects of unit price of carbon trade on emission. (**b**) The effects of unit price of carbon trade on profit.

In this section, we use a numerical example to illustrate the optimal decisions in centralized and decentralized systems. Let $d_{r}=d_{s}=200,\beta =0.01,\theta =0.5,\eta =0.005,c_{r}=10,c_{rr}=3,c_{s}=12$, $K_{r}=5000,K_{s}=8960,e_{r}=e_{s}=5,e=3,c_{e}=0-11$. According to above analysis, the total optimal profits in centralized and decentralized systems are shown in Figure 3.

From Figure 3, we can find that the higher the unit price of carbon trade is (lower than 11), the smaller the gap of total profit between centralized and decentralized systems, and the the smaller the gap of carbon emissions between centralized and decentralized systems. Obviously, the unit price of carbon trade set by government or organizations would lower than 11, in which the gap of total profit between centralized and decentralized systems is inevitable. In particular, let $c_{e}=4,8$. According to above analysis, the total optimal profits in centralized and decentralized systems are shown in Table 2.

From Table 2, we can conclude that the total profit of decentralized system is 40,327.50 (32,687.50), which is 9.42% (2.14%) lower than the profit of centralized system when the unit price of carbon trade $c_{e}=4$ ($c_{e}=8$). Hence it is necessary to coordinate supply chain to improve the performance of supply chain.

## 5. Coordination with Price Discount Contracts

To improve the performance of the dual-channel supply chain in the decentralized system, this section investigates the price discount contracts, in which the wholesale price is a discount of the channel selling price and is determined by both supplier and retailer. Since the selling price of two channels can be different, it is meaningful to compare the online channel price discount contract with the offline channel price discount contract, and find the better one to coordinate the dual-channel supply chain.

### 5.1. Online Channel Price Discount Contract

In the online channel price discount contract, the wholesale price is denoted by $w=\lambda p_{s}$, the profits of retailer and supplier are given by

(2) $$\begin{array}{ccc} \Pi_{r,s}\left(p_{r}\right) & = & D_{r}\left(p_{r}-\lambda p_{s}-c_{rr}\right)-c_{e}\left(D_{r}e_{r}-K_{r}\right) \end{array}$$

(3) $$\begin{array}{ccc} \Pi_{s,s}\left(p_{s}\right) & = & D_{s}\left(p_{s}-c_{s}\right)+D_{r}\left(\lambda p_{s}-c_{r}\right)-c_{e}\left(D_{s}e_{1}+D_{r}e-K_{s}\right) \end{array}$$

Following the analysis in Section 4.2 and substituting $w=\lambda p_{s}$ into Equation (2), we can obtain the response function of offline channel selling price

(4) $$\begin{array}{c} p_{r,s}^{*}=\frac{d_{s}\eta +\lambda A}{2A}p_{s,s}+\frac{c_{rr}+c_{e}e_{r}}{2}+\frac{d_{r}+d_{s}\left(1-\theta \right)}{2A} \end{array}$$

Substituting Equation (4) into Equation (3), solving $\frac{\partial \Pi_{s,s}}{\partial p_{s}}=0$, we can obtain the optimal selling price for given discount coefficient λ.

**Theorem** **3.** *Under the online channel price discount contract, the optimal selling prices are*

$$\begin{array}{ccc} p_{s,s}^{*}\left(\lambda \right) & = & \frac{\left[\begin{array}{c} \left(Z+d_{s}\eta \frac{d_{s}\eta \left(\lambda A-d_{s}\eta \right)}{2A}\right)c_{s}-\frac{d_{s}\eta -\lambda A}{2}\left(c_{rr}-c_{r}\right)-\frac{\lambda (A-d_{s}\eta )}{2\beta }-d_{s}\theta \\ -d_{s}\eta \frac{A-d_{s}\eta }{2A\beta }+\left[\frac{\lambda d_{s}\eta }{2}+\frac{d_{s}^{2}\eta^{2}}{2A}-d_{s}\left(\theta \beta +\eta \right)\right]c_{e}e_{1}+\left(A\lambda -d_{s}\eta \right)\frac{c_{e}\left(e_{r}-e\right)}{2} \end{array}\right]}{2\lambda d_{s}\eta -\lambda^{2}A+Z-d_{s}\left(\theta \beta +\eta \right)}\\ p_{r,s}^{*}\left(\lambda \right) & = & \frac{d_{s}\eta +\lambda A}{2A}p_{s,s}^{*}+\frac{d_{r}+d_{s}\left(1-\theta \right)}{2A}+\frac{c_{rr}+c_{e}e_{r}}{2} \end{array}$$

The online channel price discount contracts is considerable only when the profits of both supplier and retailer could increase under this contract, that is $\Pi_{s,s}^{*}>\Pi_{s}^{*}$ and $\Pi_{r,s}^{*}>\Pi_{r}^{*}$. Based on these inequalities, we will analyze the performance of online channel price discount contract by using numerical examples in Section 6.

### 5.2. Offline Channel Price Discount Contract

In the offline channel price discount contract, the wholesale price is denoted by $w=\lambda p_{r}$, the profits of retailer and supplier are given by

(5) $$\begin{array}{ccc} \Pi_{r,s}\left(p_{r}\right) & = & D_{r}\left(p_{r}-\lambda p_{r}-c_{rr}\right)-c_{e}\left(D_{r}e_{r}-K_{r}\right) \end{array}$$

(6) $$\begin{array}{ccc} \Pi_{s,s}\left(p_{s}\right) & = & D_{s}\left(p_{s}-c_{s}\right)+D_{r}\left(\lambda p_{r}-c_{r}\right)-c_{e}\left(D_{s}e_{1}+D_{r}e-K_{s}\right) \end{array}$$

From the previous analysis, solving $\frac{\partial \Pi_{r,r}}{\partial p_{r}}=0$, we can obtain the response function of offline channel selling price

(7) $$\begin{array}{c} p_{r,r}^{*}=\frac{d_{s}\eta }{2A}p_{s,r}+\frac{A-d_{s}\eta }{2A\beta }+\frac{c_{rr}+c_{r}e_{r}}{2(1-\lambda )} \end{array}$$

Substituting Equation (7) into Equation (3), solving $\frac{\partial \Pi_{s,r}}{\partial p_{s}}=0$,we can obtain the optimal selling price for given discount coefficient λ.

**Theorem** **4.** *Under the offline channel price discount contract, the optimal selling prices are*

$$\begin{array}{ccc} p_{s,r}^{*}\left(\lambda \right) & = & \frac{\left[\begin{array}{c} \left[\frac{d_{s}^{2}\eta^{2}}{2A}-d_{s}\left(\theta \beta +\eta \right)\right]\left(c_{s}+c_{e}e_{1}\right)+d_{s}\eta \frac{c_{r}+c_{e}e}{2}\\ -d_{s}\theta -\left(1+\lambda \right)\frac{d_{s}\eta \left(A-d_{s}\eta \right)}{2A\beta }-\frac{d_{s}\eta c_{rr}+c_{e}e_{r}}{2(1-\lambda )} \end{array}\right]}{\frac{\lambda d_{s}^{2}\eta^{2}}{2A}+Z-d_{s}\left(\theta \beta +\eta \right)}\\ p_{r,r}^{*}\left(\lambda \right) & = & \frac{d_{s}\eta }{2A}p_{s,r}^{*}+\frac{A-d_{s}\eta }{2A\beta }+\frac{c_{rr}+c_{e}e_{r}}{2(1-\lambda )} \end{array}$$

The online channel price discount contracts is considerable only when the profits of both supplier and retailer could increase under this contract, that is $\Pi_{s,r}^{*}>\Pi_{s}^{*}$ and $\Pi_{r,r}^{*}>\Pi_{r}^{*}$. Based on these inequalities, we will analyze the performance of online channel price discount contract by using numerical examples in Section 6.

### 5.3. Comparison of Two Channel Price Discount Contracts

When both the online channel and offline channel price discount contracts can generate higher profits for the supplier and retailer, we provide the following approach to compare the coordination effects of the two price discount contracts.

As mentioned in Section 5.1 and Section 5.2, if the online channel and offline channel price discount contracts can generate higher profits for the supplier and retailer, we have

$$\begin{array}{c} \Pi_{r,s}^{*}\left(\lambda \right)>\Pi_{r}^{*},\;\Pi_{s,s}^{*}\left(\lambda \right)>\Pi_{s}^{*};\\ \Pi_{r,r}^{*}\left(\lambda \right)>\Pi_{r}^{*},\;\Pi_{s,r}^{*}\left(\lambda \right)>\Pi_{s}^{*}. \end{array}$$

Then, we can calculate the profit changes for the supplier and retailer under the two channel price discount contracts, that is,

$$\begin{array}{c} \Delta_{r,s}=\Pi_{r,s}^{*}\left(\lambda_{s}\right)-\Pi_{r}^{*},\;\Delta_{s,s}=\Pi_{s,s}^{*}\left(\lambda_{s}\right)-\Pi_{s}^{*};\\ \Delta_{r,r}=\Pi_{r,r}^{*}\left(\lambda_{r}\right)-\Pi_{r}^{*},\;\Delta_{s,r}=\Pi_{s,r}^{*}\left(\lambda_{r}\right)-\Pi_{s}^{*}. \end{array}$$

If $\Delta_{r,s}>\Delta_{r,r}>0$ and $\Delta_{s,s}>\Delta_{s,r}>0$, we conclude that the online channel price discount contract is better than the offline channel price discount contract. If $0<\Delta_{r,s}<\Delta_{r,r}$ and $0<\Delta_{s,s}<\Delta_{s,r}$, we conclude that the offline channel price discount contract is better than the online channel price discount contract. Otherwise, it is difficult to say which price discount contract is better. Because of the complexity of the mathematical expressions, we will investigate the performance of the two price discount contracts through numerical experiments in the next section.

## 6. Numerical Example

In this section, we use numerical experiments to illustrate the coordination effect for two kinds of price discount contracts. The basic parameters of the example as follows $d_{r}=d_{s}=2000$, β=0.01, θ=0.5, $\eta =0.005,c_{r}=10,c_{rr}=3,c_{s}=12,K_{r}=1000,K_{s}=2500,e_{r}=e_{s}=5,e=3,c_{e}=4,8$. The calculation results of centralized and decentralized systems before coordination is in Table 2 in Section 4.3. When $c_{e}=4$, the profits supplier and retailer are $\Pi_{s}^{*}=32,125.00,\Pi_{r}^{*}=8202.50$ and the profit of supply chain system is 40,327.50, while the profit under centralized system is 44,522.50. When $c_{e}=8$, the profits supplier and retailer are $\Pi_{s}^{*}=23,965.00,\Pi_{r}^{*}=8722.50$ and the profit of supply chain system is 32,687.50, while the profit under centralized system is 33,402.50.

**Figure 4:**
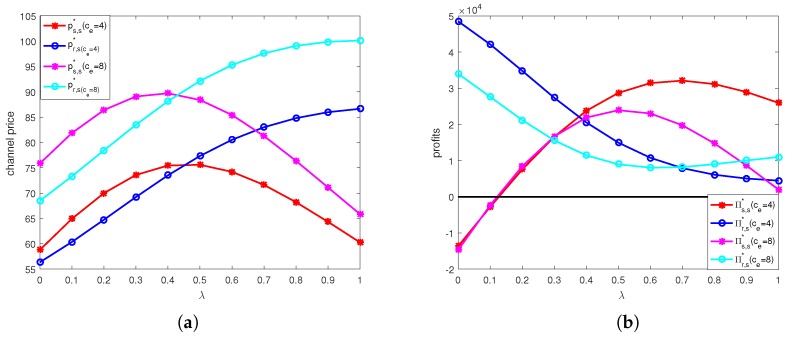
Optimal selling price and profit under online channel price discount contract: (**a**) Optimal selling price. (**b**) Optimal profit.

**Figure 5:**
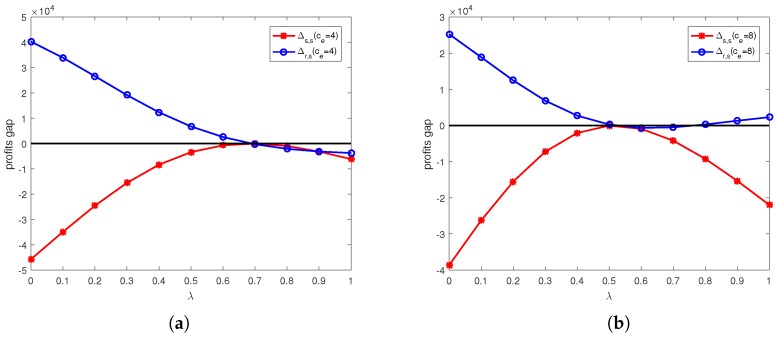
The profit gap before and after online channel price discount contracts under different unit price of carbon trade: (**a**) $c_{e}=4$. (**b**) $c_{e}=8$.

**Figure 6:**
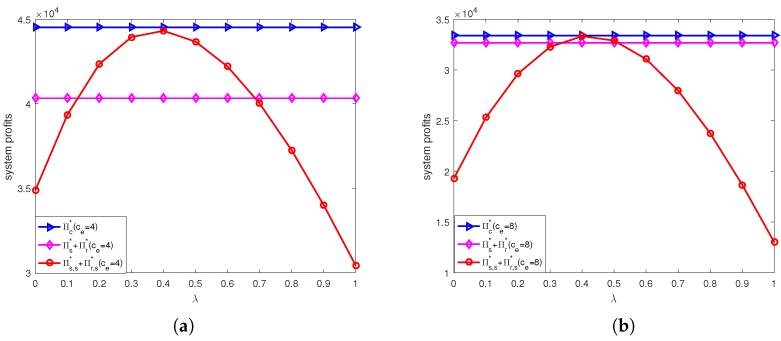
Profit comparison before and after online channel price discount contracts under different unit price of carbon trade: (**a**) $c_{e}=4$. (**b**) $c_{e}=8$.

**Figure 7:**
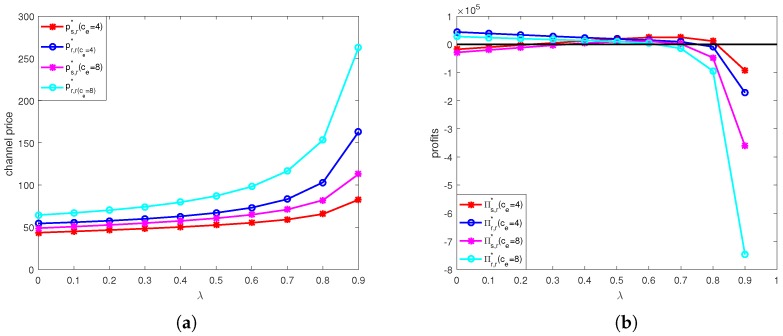
Optimal selling price and profit in offline channel supply chain: (**a**) Optimal selling price in online channel supply chain. (**b**) Optimal profit in offline channel supply chain.

The calculation results under online channel and offline channel price discount contracts are shown in Figure 4, Figure 5, Figure 6 and Figure 7.

Figure 4 shows the effects of unit price of carbon trade and discount coefficient on retail prices of two channels and profits of supplier and retailer under online channel price discount contract. From Figure 4a, we can see that under the online price discount contract, the selling prices of both online channel and offline channel increase as λ increases when λ<0.4. When λ≥0.4, the online channel selling price decreases as λ increases, while the offline channel selling price increases. We also find that the selling prices of both two channels when $c_{e}=4$ are lower than those when $c_{e}=8$, that is higher unit price of carbon trade would lead to higher selling prices. From Figure 4b, we can see that the supplier’s profit first increases and then decreases when λ≥0.7 with unit price of carbon trade, and the profit of retailer always decreases when $c_{e}=4$, while when the unit price of carbon trade $c_{e}=8$, the supplier’s profit first increases and then decreases when λ≥0.5, and retailer’s profit would first decrease and then increase when λ≥0.6. This means that higher unit price of carbon trade would change the trend of profit in advance.

Figure 5 shows the effects of unit price of carbon trade and discount coefficient on profit changes before and after online channel price discount contract. From Figure 5a, we can see that when $c_{e}=4$, the retailer’s profit higher than that in decentralized system before coordination when λ<0.7, and supplier’s profit lower than that in decentralized system before coordination in online channel price discount contract. That is the profits of retailer and supplier both lower than that in decentralized system before coordination when λ≥0.7. Therefore the retailer would like to accept the online channel price discount contract when λ<0.7. It means there exist a divergence on using this contract. From Figure 5b, we can see that when $c_{e}=8$, the retailer’s profit higher than that in decentralized system before coordination when λ<0.5 or λ>0.75, and and supplier’s profit lower than that in decentralized system before coordination in online channel price discount contract. Therefore the retailer would like to accept the online channel price discount contract when λ<0.5 or λ>0.75. It means there exist a divergence on using this contract.

Figure 6 shows the coordination performance of online channel price discount contract under different unit price of carbon trade. From Figure 6a, we can see that when $c_{e}=4$, the profit of decentralized system in online channel price discount is higher than that without coordination when 0.12<λ<0.69, it means that this contract could improve supply chain performance; in particular, the system profit after coordination almost achieves the profit in the centralized system when λ=0.4. From Figure 6b, we can see that when $c_{e}=8$, the profit of decentralized system in online channel price discount is higher than that without coordination when 0.35<λ<0.5; in particular, the system profit after coordination almost achieves the profit in the centralized system when λ=0.4. We also find that higher unit price of carbon trade reduces the scope of online channel price discount contract to achieve coordination. It means that it is difficult for online channel price discount contract to achieve coordination with a higher unit price of carbon trade.

However, we should note that even though the online channel price discount contract can achieve perfect coordination with unit price of carbon trade $c_{e}=4$ when λ=0.4, and the retailer prefer to online channel price discount contract, while the supplier does not would like to accept this contract (Figure 5a). To implement this contract, the retailer could design a transfer payment mechanism to both improving profits of retailer and supplier. When λ=0.4, $\Delta_{s,s}=-8355.00$, $\Delta_{r,s}=12,347.00$. If the transfer payment mechanism could satisfy certain conditions, both sides could achieve win-win. Due to the retailer’s profit in contract increases, it would like to pay certain extra expense to supplier. This shows the feasibility of transfer payment, which range (8355.00, 12,347.00). This mechanism make the increased profit redistribution after supply chain coordination and the amount of distribution depends on the bargaining power of both sides. Similarly, when $c_{e}=8$ the online channel price discount contract can achieve perfect coordination when λ=0.4, and the retailer prefer to online channel price discount contract, while the supplier does not would like to accept this contract (Figure 5b). Here, $\Delta_{s,s}=-2068.60$, $\Delta_{r,s}=2715.50$, then the retailer should pay certain extra expense to supplier, which is (2068.60, 2715.50).

Figure 7 show the effects of of unit price of carbon trade and discount coefficient on retail prices of two channels and profits of supplier and retailer under offline channel price discount contract. From Figure 7a, we can see that under the offline price discount contract, the selling prices of both online channel and offline channel increase as λ increases. We also find that the selling prices of both two channels when $c_{e}=4$ are lower than those when $c_{e}=8$, that is higher unit price of carbon trade would lead to higher selling prices. From Figure 7b, we can see that the supplier’s profit first increases and then decreases when λ≥0.7, and the profit of retailer always decreases, and the profits of retailer and supplier are higher when $c_{e}=4$ than those when $c_{e}=8$. This means that higher unit price of carbon trade would reduce profits of retailer and supplier in offline channel price discount contract.

**Figure 8:**
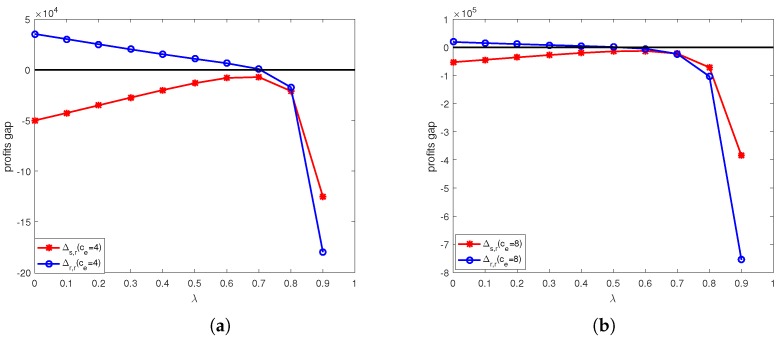
The profit gap before and after offline channel price discount contracts under different unit price of carbon trade: (**a**) $c_{e}=4$. (**b**) $c_{e}=8$.

Figure 8 shows the effects of unit price of carbon trade and discount coefficient on profit changes before and after offline channel price discount contract. From Figure 8a, we can see that when $c_{e}=4$, the retailer’s profit higher than that in decentralized system before coordination when λ<0.7, and supplier’s profit lower than that in decentralized system before coordination in offline channel price discount contract. That is the profits of retailer and supplier both lower than that in decentralized system before coordination when λ≥0.7. Therefore the retailer would like to accept the online channel price discount contract when λ<0.7. It means there exist a divergence on using this contract. From Figure 8b, we can see that when $c_{e}=8$, the retailer’s profit higher than that in decentralized system before coordination when λ<0.4, and and supplier’s profit lower than that in decentralized system before coordination in offline channel price discount contract. Therefore the retailer would like to accept the online channel price discount contract when λ<0.4. It means there exist a divergence on using this contract.

**Figure 9:**
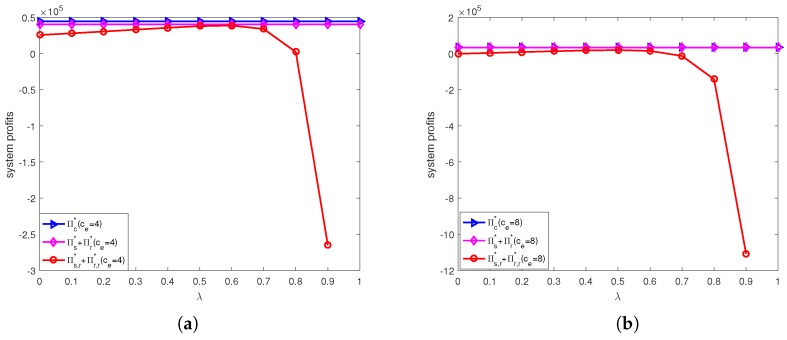
Profit comparison before and after offline channel price discount contracts under different unit price of carbon trade: (**a**) $c_{e}=4$. (**b**) $c_{e}=8$.

Figure 9 shows shows the coordination performance of online channel price discount contract under different unit price of carbon trade. We can find that the system profit in offline channel price discount contract lower than that in decentralized system before coordination. It means that this contract does not work in improving supply chain performance, so we should not use it to coordinating supply chain.

## 7. Conclusions

This paper studies a two-echelon dual-channel supply chain under a cap-and-trade regulation. When demand is influenced by channel prices, it is more difficult to determine the optimal price for each channel and the best wholesale price for the retail channel. We first analyze the optimal decisions in centralized and decentralized systems under the Stackelberg game and we obtain the optimal pricing decisions and corresponding profits. We explore the effects of unit price of carbon trade on decision making and corresponding profits of supplier and retailer. Moreover, we numerically find that there is a system profit gap between centralized and decentralized systems, and we provide two kinds of price discount contracts to coordinate the dual-channel supply chain under cap-and-trade regulation. We also show the effects of carbon regulation on coordination performance. Numerical example shows that the online channel price discount contract could coordinate the dual-channel supply chain under cap-and-trade regulation and it just needs a transfer payment mechanism to redistribution the increased profit to make win-win of two sides. At this moment the profit in decentralized system almost equals to that of centralized system.

Several important issues are retained for future research. First, the pricing and coordination of dual-channel supply chain with a random demand under cap-and-trade regulation will be an interesting research. Second, we could explore the competitive equilibrium of the dual-channel supply chain under cap-and-trade regulation in asymmetric information settings.

## Figures and Tables

**Table:** 

(**a**)	(**b**)

**Table:** 

(**a**)	(**b**)

**Table:** 

(**a**)	(**b**)

**Table:** 

(**a**)	(**b**)

**Table:** 

(**a**)	(**b**)

**Table:** 

(**a**)	(**b**)

**Table:** 

(**a**)	(**b**)

**Table:** 

(**a**)	(**b**)

**Table 1:** Major notations and explanations.

Notation	Explanation
For system	
$d_{r}$	Market size composed of retailer loyal customers
$d_{s}$	Market size composed of brand loyal customers
$K_{c}$	Carbon emission capacity assigned to centralized system, where $K_{c}=K_{r}+K_{s}$
$\Pi_{c}\left(\cdot \right)$	Total profit of dual-channel supply chain in centralized system
λ	Price discount coefficient in the price discount contract
$c_{e}$	Unit price of carbon trade
for the supplier	
*w*	Unit wholesale price to retailer(decision variable)
$p_{s}$	Unit selling price in online channel (decision variable)
$c_{r}$	Retailer’s unit order cost for the product in offline channel
$c_{s}$	Supplier’s unit order cost for the product in online channel
*e*	Carbon emission of unit product generated during the supplier’s production
$e_{s}$	Carbon emission of unit product generated during procurement
$K_{s}$	Carbon emission capacity assigned to supplier
$\Pi_{s}\left(\cdot \right)$	Supplier’s total profit in decentralized system
$\Pi_{s,r}\left(\cdot \right)$	Supplier’s total profit under the offline channel price discount contract
$\Pi_{s,s}\left(\cdot \right)$	Supplier’s total profit under the online channel price discount contract
for the retailer	
$p_{r}$	Unit selling price in offline channel (decision variable)
$c_{rr}$	Unit operational cost of retailer
$e_{r}$	Carbon emission of unit product in offline channel procurement
$K_{r}$	Carbon emission capacity assigned to retailer
$\Pi_{r}\left(\cdot \right)$	Total profit of retailer in decentralized system
$\Pi_{r,r}\left(\cdot \right)$	Total profit of retailer under offline channel price discount contract
$\Pi_{r,s}\left(\cdot \right)$	Total profit of retailer under online channel price discount contract

**Table 2:** The total profits in centralized and decentralized systems.

Notations	Centralized System	Decentralized System
$p_{s}$	(72.00, 88.00)	(72.00, 88.00)
$p_{r}$	(72.50, 88.50)	(82.75, 92.75)
*w*	( –, –)	(49.50, 45.50)
$D_{s}$	(285.00, 125.00)	(387.50, 167.50)
$D_{r}$	(820.00, 340.00)	(410.00, 170.00)
$\Pi_{s}$	( –, –)	(32,125.00, 23,965.00)
$\Pi_{r}$	( –, –)	(8202.50, 8722.50)
$\Pi_{s}+\Pi_{r}$	(44,522.50, 33,402.50)	(40,327.50, 32,687.50)

Please note that (·,·) is a vector of the solutions in two cases ‘$c_{e}=4$’ and ‘$c_{e}=8$’.

## Appendix A

It is easy to verify that total profit of supply chain $\Pi_{c}\left(p_{r},p_{s}\right)$ is a convex programming about $p_{r}$ and $p_{s}$.

Let $\frac{\partial \Pi_{c}\left(p_{r},p_{s}\right)}{\partial p_{r}}=0$ and $\frac{\partial \Pi_{c}\left(p_{r},p_{s}\right)}{\partial p_{s}}=0$, we can obtain

$$\begin{array}{ccc} \frac{\partial \Pi_{c}\left(p_{r},p_{s}\right)}{\partial p_{r}} & = & -2\beta \left(d_{r}+d_{s}\left(1-\theta \right)\right)p_{r}-2d_{s}\eta p_{r}+2d_{s}\eta p_{s}+\left(d_{r}+d_{s}\left(1-\theta \right)\right)\beta \left(c_{r}+c_{r}r+c_{e}e_{2}+1\right)\\ & & +d_{s}\eta \left(c_{r}+c_{rr}-c_{s}-c_{e}e_{1}+c_{e}e_{2}\right)=0\\ \frac{\partial \Pi_{c}\left(p_{r},p_{s}\right)}{\partial p_{s}} & = & -2\left(d_{s}\theta \beta +d_{s}\eta \right)p_{s}+2d_{s}\eta p_{r}+d_{s}\theta \beta \left(c_{s}+c_{e}e_{1}\right)\\ & & -d_{s}\eta \left(c_{r}+c_{rr}-c_{s}-c_{e}e_{1}+c_{e}e_{2}\right)=0 \end{array}$$

Then we have $\frac{\partial^{2}\Pi_{c}\left(p_{r},p_{s}\right)}{\partial p_{r}^{2}}=-2\beta \left(d_{r}+d_{s}\left(1-\theta \right)\right)-2d_{s}\eta <0$, $\frac{\partial^{2}\Pi_{c}\left(p_{r},p_{s}\right)}{\partial p_{s}^{2}}=-2\left(d_{s}\theta \beta +d_{s}\eta \right)<0$, and the Hessian Matrix is negative definite.

## Appendix B

We can obtain above results by solving simultaneous equations $\frac{\partial \Pi_{c}\left(p_{r},p_{s}\right)}{\partial p_{r}}=0$ and $\frac{\partial \Pi_{c}\left(p_{r},p_{s}\right)}{\partial p_{s}}=0$.

## Appendix C

Substituting Equation (1) to $\Pi_{s}\left(p_{s},w\right)$, we can find that $\Pi_{s}\left(p_{s},w\right)$ is a strictly convex function about $p_{s}$ and *w*.

Let $\frac{\partial \Pi_{s}\left(p_{s},w\right)}{\partial p_{s}}=0$ and $\frac{\partial \Pi_{s}\left(p_{s},w\right)}{\partial w}=0$, we can obtain

$$\begin{array}{ccc} \frac{\partial \Pi_{s}\left(p_{s},w\right)}{\partial p_{s}} & = & \left(\frac{d_{s}^{2}\eta^{2}}{A}-2d_{s}\left(\theta \beta +\eta \right)\right)p_{s}+\frac{d_{s}^{2}\eta^{2}}{2A}-d_{s}\left(\theta \beta +\eta \right)\left(c_{s}+c_{e}e_{1}\right)+d_{s}\theta \\ & & +d_{s}\eta \frac{d_{r}+d_{s}\left(1-\theta \right)}{2A}+d_{s}\eta \frac{2w+c_{rr}-c_{r}+c_{e}e_{r}-c_{e}e}{2}=0\\ \frac{\partial \Pi_{s}\left(p_{s},w\right)}{\partial w} & = & -A\frac{2w+c_{rr}-c_{r}+c_{e}e_{r}-c_{e}e}{2}+d_{s}\eta p_{s}+\frac{d_{r}+d_{s}\left(1-\theta \right)-d_{s}\eta \left(c_{s}+c_{e}e_{r}\right)}{2}=0 \end{array}$$

Then we have $\frac{\partial^{2}\Pi_{s}\left(p_{s},w\right)}{\partial p_{s}^{2}}=\frac{d_{s}^{2}\eta^{2}}{A}-2d_{s}\left(\theta \beta +\eta \right)<0$, $\frac{\partial^{2}\Pi_{s}\left(p_{s},w\right)}{\partial w^{2}}=-A<0$, and the Hessian Matrix is negative definite.

## Appendix D

We can obtain above results by solving Equation (1),$\frac{\partial \Pi_{s}\left(p_{s},w\right)}{\partial p_{s}}=0$ and $\frac{\partial \Pi_{s}\left(p_{s},w\right)}{\partial w}=0$.
